# Single-Channel Blind Signal Separation of the MHD Linear Vibration Sensor Based on Singular Spectrum Analysis and Fast Independent Component Analysis

**DOI:** 10.3390/s22249657

**Published:** 2022-12-09

**Authors:** Mengjie Xu, Jianhan Wang, Jiahui Mo, Xingfei Li, Lei Yang, Feng Ji

**Affiliations:** 1 School of Instrument Science and Opto-Electronics Engineering, Hefei University of Technology, Hefei 230009, China; 2 State Key Laboratory of Precision Measuring Technology and Instruments, Tianjin University, Tianjin 300072, China

**Keywords:** magnetohydrodynamic linear vibration sensor, singular spectrum analysis, fast independent component analysis

## Abstract

An MHD vibration sensor, as a new type of sensor used for vibration measurements, meets the technical requirements for the low-noisy measurement of acceleration, velocity, and micro-vibration in spacecraft during their development, launch, and orbit operations. A linear vibration sensor with a runway type based on MHD was independently developed by a laboratory. In a practical test, its output signal was mixed with a large amount of noise, in which the continuous narrowband interference was particularly prominent, resulting in the inability to efficiently carry out the real-time detection of micro-vibration. Considering the high interference of narrowband noise in linear vibration signals, a single-channel blind signal separation method based on SSA and FastICA is proposed in this study, which provides a new strategy for linear vibration signals. Firstly, the singular spectrum of the linear vibration signal with noise was analyzed to suppress the narrowband interference in the collected signal. Then, a FastICA algorithm was used to separate the independent signal source. The experimental results show that the proposed method can effectively separate the useful linear vibration signals from the collected signals with low SNR, which is suitable for the separation of the MHD linear vibration sensor and other vibration measurement sensors. Compared with EEMD, VMD, and wavelet threshold denoising, the SNR of the separated signal is increased by 10 times on average. Through the verification of the actual acquisition of the linear vibration signal, this method has a good denoising effect.

## 1. Introduction

With the development of space technology, the field of space engineering demands increasingly high standards for the structural design of spacecraft, among which, the analysis of the mechanical environmental conditions of spacecraft is key to its overall design [1]. The mechanical environment of spacecraft refers to the environment of vibration, acceleration, noise, and microgravity that the spacecraft is subjected to during operation. Wide-band and low-noise vibration sensors can meet the needs of the wide-band, high-precision mechanical environment testing of spacecraft [2].

In this paper, a gallium–indium–tin alloy with good conductivity and no bias is used as the conductive fluid material for the MHD linear vibration sensors. A linear vibration sensor based on the MHD effect has the advantages of a high yield strength, good dynamic performance, and no mechanical wear phenomenon between internal firmware. It can be easily used in the scenarios of wide-band frequency and high-precision measurements, and can be used for effective in-orbit space vibration measurements. In the actual vibration measurement, the measurement system contains multiple error sources inside, and the weak broadband signals detected by the MHD linear vibration sensors are often mixed with multiple noise sources. At the same time, the lack of accuracy in the dynamic measurement process remains a key concern for engineering measurement personnel and instrument designers. We need to take effective and efficient measures to eliminate or reduce interference information, and thus improve the accuracy of the dynamic measurement system.

The requirement for the MHD linear vibration sensors in aerospace technology to have amplitudes within millivolts and vibration frequencies within kilohertz, coupled with the complex external environment [1], makes signal acquisition and extraction separation difficult. Therefore, the extraction and separation of weak signals output from the MHD linear vibration sensors is one of the key techniques used to perform micro-vibration measurements [3].

Blind source separation (BSS) is a blind signal extraction technology, which is often used to separate and extract independent sources in mixed signals [4,5,6]. Independent component analysis (ICA), as a blind source separation technology, is widely used to separate covered source signals from mixed signals [7]. An independent component analysis cannot directly solve the underdetermined blind source separation problem of the MHD line vibration sensor output signal presented in this paper. Single-channel signals can be converted into multi-channel signals by some methods, and then the converted multi-channel signals can be used for ICA [8]. For example, in 2016, Lu et al. proposed a new wavelet threshold function and its denoising application [9]. From the SNR comparison of the processed signals, using this method it can be seen that the SNR of the output signal is the largest; 1–2.7 times higher than that of the traditional threshold denoising method. In 2018, Ajay Kumar Maddirala and Rafi Ahamed Shaik proposed a single-channel EEG signal source separation method combining SSA and ICA [10]. Through the simulation of analog signals, it was verified that this method can decompose the hidden source in a single-channel EEG signal and has a high source separation efficiency. In 2019, Meng Zhou et al. proposed a photoacoustic sensing imaging denoising algorithm based on empirical mode decomposition (EMD) and ICA [11], which can be applied to most pulse signal denoising problems. In the same year, Tao Jin et al. proposed a novel adaptive integrated empirical mode decomposition (EEMD) method based on partial discharge [12] and applied it to the simulated pulse signal. This method has a good denoising effect, and the SNR improves by about 1.2 times. In 2020, Dariusz Mika et al. proposed a single-channel source separation algorithm based on ICA time frequency decomposition [13], which used an ICA method to separate single-channel source signals from single mixed signals. The main limitation of this method is the lack of generality. The choice of distance measure and clustering algorithm depends on the time frequency structure of the mixed signal. As the number of mixed signals increases, the quality of the signal of separated components decreases. In 2020, Liu et al. proposed the vibration signal analysis of water seal blasting based on wavelet threshold denoising and HHT transform [14]. In 2022, Yufei Zhou et al. proposed an improved single-channel BSS algorithm based on variational mode decomposition (VMD) [15]. This algorithm is applied to the simulated signal, which effectively avoids the endpoint effect and mode mixing, and makes decomposition more stable. The decomposition layers of different signals are determined by setting different spectral correlation coefficient thresholds, and the sparse frequency division of the received mixed signals is realized. In summary, modal aliasing occurs when the signals are decomposed by EMD [16,17,18]. EEMD can solve the pattern aliasing phenomenon in the EMD method [19,20,21,22], but it needs to increase the ensemble average to compensate for the added white noise. White noise and the reference signal are not easy to apply when a real-time vibration measurement is carried out by an MHD linear vibration sensor. Moreover, the EMD-like method is used to process the output signal of the MHD linear vibration sensor, with a long calculation time and large reconstruction error, so the processed signal is likely to have a low SNR. Therefore, the EMD-like method is not suitable for processing the output signal of an MHD linear vibration sensor.

In order to separate independent source signals from mixed signals with a low SNR, this paper proposes a method of MHD linear vibration signal separation based on SSA and FastICA. In this paper, SSA was first used to suppress the periodic narrowband interference and white noise in an acquired signal, and a noise-reduced mixed acceleration signal was obtained. Then, the noise-reduced signal was separated by a modified, fast, independent component analysis to obtain an unmixed signal. The unmixed signal was analyzed and identified to determine the useful and noisy components of the acquired magnetohydrodynamic sensor signal. Experimental analysis shows that this method can achieve the effective separation of independent line vibration signals with reasonable suppression of linear vibration signal admixture noise.

In the second section of this paper, the working principle of the MHD linear vibration sensor is described. In the third section, the blind source separation algorithm based on SSA and FastICA is proposed. In the fourth section, the experimental results are compared and verified.

## 2. The Working Principle of the MHD Linear Vibration Sensor

**Figure 1:**
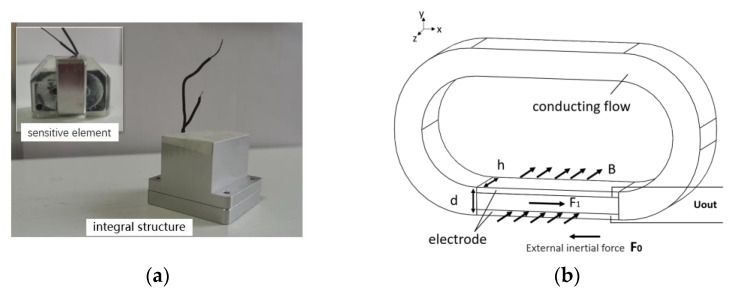
(**a**) The physical map of the quartz flexible accelerometer; (**b**) schematic diagram of the MHD linear vibration sensor principle.

When the MHD linear vibration sensor is functioning, the conductive fluid moves in a circle in the magnetic field inside the channel. Figure 1(a) shows the structure diagram of the MHD linear sensor. The principle of the MHD linear vibration sensor is shown in Figure 1(b), where h is the height of the channel, d is the thickness of the channel, the entire working area is in a constant magnetic field B, F_0_ is the external inertia force on the measured carrier, and F_1_ is the Lorentz force on the conducting fluid in the magnetic field. Based on the principle of electromagnetic induction, the conductive fluid moves along the cutting magnetic inductive v under the superposition of inertia force and Lorentz force. According to the laws of electromagnetic induction, i.e., mechanical energy into electrical energy, due to the upper and lower wall conduction, an electric potential difference between the two walls is produced on the MHD channel between the internal and external wall, and the relative velocity and magnetic induction intensity forms a certain relationship between motional and electromotive force U_out_, U_out_ *=* v·B (v is the relative velocity between the conductive fluid and the wall of the fluid channel).

**Figure 2:**
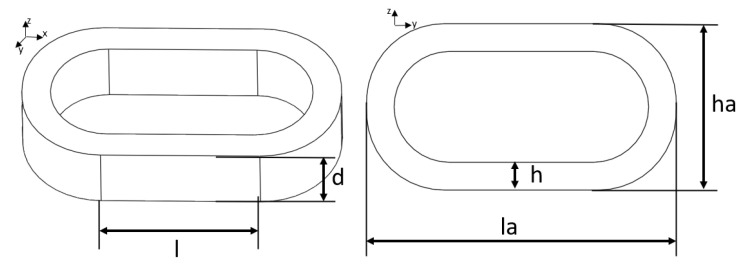
Schematic diagram of basic dimensions of the MHD linear vibration sensor fluid channel.

According to the working principle of the MHD linear vibration sensor, the input–output model of the sensor was theoretically derived. The MHD explores the interaction between the motion field of conductive fluid and the magnetic field of the medium. It not only observes the influence of the electromagnetic field on the motion of conductive fluid, but also explores the reaction of fluid motion on the electromagnetic field. Without considering the influence of temperature change, according to the theory of MHD, the motion of Newtonian fluid and the basic size diagram of the sensor fluid channel are combined, as shown in Figure 2.

Without considering the influence of temperature change, according to the theory of MHD [23], by combining continuity equation and Navier–Stokes equation, we can obtain:

(1) $$\frac{\partial v}{\partial t}+(v\cdot \nabla )v=-\frac{1}{\rho }\nabla p_{f}+\mu \nabla^{2}v-\frac{1}{\rho }J\times B+a$$

where ρ is the fluid density, $p_{f}$ is the pressure, *μ* is the dynamic viscosity, F_1_ = −*J* × *B* (F_1_ is the Lorentz force [24]), *a* is acceleration in the inertial force F_0_ (F_0_ = *m* × *a*).

In the coordinate system, expanding along the *X*-axis, the main direction of movement is as follows:

(2) $$\frac{\partial v_{x}}{\partial t}+\left(v_{x}\cdot \frac{\partial v_{x}}{\partial x}+v_{y}\cdot \frac{\partial v_{x}}{\partial y}+v_{z}\cdot \frac{\partial v_{x}}{\partial z}\right)=-\frac{1}{\rho }\cdot \frac{\partial p_{f}}{\partial x}+\mu \left(\frac{\partial^{2}v_{x}}{\partial x^{2}}+\frac{\partial^{2}v_{x}}{\partial y^{2}}+\frac{\partial^{2}v_{x}}{\partial z^{2}}\right)-\frac{1}{\rho }J\times B+a$$

The following assumptions are made about the sensor during the model construction:

(1) Ignoring Secondary Flow Assumption:

In order to minimize the impact of secondary flows, the following should be carried out: $v_{y}\ll v_{x}$, $v_{z}\ll v_{x}$.

(3) $$\left(v_{x}\cdot \frac{\partial v_{x}}{\partial x}+v_{y}\cdot \frac{\partial v_{x}}{\partial y}+v_{z}\cdot \frac{\partial v_{x}}{\partial z}\right)\sim \left(v_{x}\cdot \frac{\partial v_{x}}{\partial x}\right)$$

(2) Poiseuille Flow Hypothesis:

Assuming that the fluid flow in the working area along the X direction is a time-invariant one-dimensional linear flow [25], and conforms to the Hartmann flow in the two-dimensional straight channel, the Navier–Stokes equation and the magnetic diffusion equation need to be used. Since the coupling between the velocity vector and the magnetic field in the MHD can be expressed through Maxwell’s equations [26,27], the magnetic diffusion equation is composed of Maxwell’s equations, which are transformed by Faraday’s law, Ohm’s law, and Ampere’s law through differentiation, and its expression form is as follows:

(4) $$\frac{\partial B}{\partial t}=\nabla \times \left(v\times B\right)+\eta \nabla^{2}B$$

In the working area, the conductive fluid moving in the *X*-axis direction will have a flow velocity distribution in the *Z*-axis direction, which follows the law of the Poisson lobe flow between the two plates [28], and its steady-state solution flow velocity is trapezoidal.

When $z=\pm k\left(k=1/2h,h=h_{2}-h_{1}\right)$, $v_{x}=0$, and since $V=v_{x}-v$, the maximum velocity in X direction can be deduced as follows:

(5) $$V_{xf}=-\frac{1}{2}\mu_{f}\frac{\partial p_{f}}{\partial x}\left(z^{2}-k^{2}\right)=\left(v_{x}-v\right)\left(1-\frac{z^{2}}{k^{2}}\right)$$

(3) Assumption from Ignoring the Induced Magnetic Field:

When the magnetic Reynolds number of the model is small, the magnetic field generated by the internal induced current (induced magnetic field) is much smaller than that of the external applied magnetic field. Therefore, the generation of the coupled magnetic field is not considered here, and the induced magnetic field is ignored.

(4) Ideal Fluid Hypothesis:

The conductive fluid is assumed to be an ideal fluid with a small viscosity and incompressibility. According to Bernoulli’s equation, we can conclude that the sum of the pressure of potential energy, upper potential energy, and kinetic energy is equal to the constant.

By partial differential treatment, the following results can be obtained:

(6) $$\frac{\partial p_{f}}{\partial x}=-\frac{1}{2}\rho \cdot 2v_{x}\cdot \frac{\partial v_{x}}{\partial x}=-\rho v_{x}\cdot \frac{\partial v_{x}}{\partial x}$$

Combined with the above assumptions, the Navier–Stokes equation can be simplified as follows:

(7) $$\frac{\partial v_{x}}{\partial t}+\frac{v_{x}}{l}=-\frac{1}{\rho }\left(-\rho dh\cdot \frac{v_{x}}{l}\right)-\mu \frac{v_{x}-v}{h^{2}}-\frac{1}{\rho }\sigma \cdot B^{2}\cdot v+a$$

The simplified Navier–Stokes equations are obtained by the Laplace transformation as:

(8) $$a(s)=\frac{s^{2}+\left(\frac{\mu }{h^{2}}+\frac{1-dh}{l}\right)s}{s+\frac{\mu }{h^{2}}+\frac{\sigma B^{2}}{\rho }}v_{x}(s)$$

From the law of electromagnetic induction, the formula for the induced electric potential can be derived as:

(9) $$U=\int_{0}^{k}Bv_{x}dh$$

Therefore, the transfer function of the runway-type fluid ring linear vibration sensor based on the MHD is:

(10) $$\left|\frac{U\left(s\right)}{V_{out}\left(s\right)}\right|=\frac{Bh\left(s+\frac{\mu }{h^{2}}+\frac{\sigma B^{2}}{\rho }\right)}{s+\left(\frac{\mu }{h^{2}}+\frac{1-dh}{\rho l}\right)}$$

## 3. Single-Channel Blind Signal Separation Based on SSA and FastICA

### 3.1. Principle of SSA

Based on subspace technology [29], SSA can identify and enhance the characteristics of periodic signals in a time series without prior information, and is not subject to the advantages of sine wave constraints. It combines the empirical orthogonal function to reconstruct the time series, and can extract the signal from the time series data with limited length, which is especially suitable for studying the periodic oscillation behavior of a time series. Currently, SSA is also widely used to decompose single-channel signals into trend, oscillation, and noise components.

A singular analysis is based on the practice of constructing the observed sequence path matrix, and the trajectory of matrix decomposition and reconstruction. The aim is to extract signals on behalf of the original time series signal of different components, such as long-term trend, and periodic and noise signals, and the structure of time series analysis can be used for further predictions. In this paper, SSA denoising actually disregards the main components of the original data and considers the unimportant components as noise, so as to remove this noise. The basic steps are to first arrange the original sequence into a matrix form with a delay, construct the trajectory matrix, and then use singular value decomposition. Then, a new matrix formed by the obtained principal components is grouped and diagonally averaged [19]. The main calculation steps of SSA technology are given as follows:

(1) Decomposition

The decomposition stage consists of two sub-steps, namely embedding and SVD. In the embedding step, the N sampled signal vectors *y* = [*y*(1), *y*(2), …, *y*(N)] are mapped into the *M* × *L* trajectory matrix *Y*:

(11) $$Y=\left[\begin{array}{cccc} y\left(1\right) & y\left(2\right) & \cdots & y\left(L\right)\\ y\left(2\right) & y\left(3\right) & \cdots & y\left(L+1\right)\\ \vdots & \vdots & \ddots & \vdots \\ y\left(M\right) & y\left(M+1\right) & \cdots & y\left(L+M\right) \end{array}\right]$$

The trajectory matrix Y is called the Hankel matrix, and the value of each element is equal to the value of the adjacent elements in the diagonal direction of the matrix. Where M is the decomposition length, its value can be flexibly selected according to the actual processing of the signal, usually not more than half of the signal length.

In the SVD step, a singular value decomposition (SVD) is performed on the trajectory matrix *Y,* that is, *Y* = UΣV^T^, where U is called the left matrix, ∑ only has values on the main diagonal, which is the singular value, and all other elements are zero. V represents the right matrix. In addition, both U and V are unit orthogonal matrices, which satisfy UU^T^ = E, VV^T^ = E. As it is difficult to directly decompose the trajectory matrix, the covariance matrix of the trajectory matrix, S *= YY^T^*, was first calculated and then the eigenvalue decomposition of S was carried out to obtain eigenvalues λ_1_ > λ_2_ > … > λ_L_ ⩾ 0 and the corresponding eigenvectors U_1_, U_2_, …, U_n_; then, U = [U_1_, U_2_, …, U_L_], λ_1_^1/2^ > λ_2_^1/2^ > … > λ_L_^1/2^ = 0 represents the singular spectrum of the original sequence.

(2) Reconstruction

Based on the singular value decomposition (SVD), the original time series are divided into several disjoint groups, and the original data are divided into several series according to their degree of importance, so as to prepare for the next reconstruction. Finally, the first M (decomposition length) main components are selected to form a new time series to achieve reconstruction.

### 3.2. FastICA Algorithm

FastICA is a fast optimization iterative algorithm derived from the principle of non-Gaussianity maximization [30]. Considering that an algorithm to maximize the objective function is needed in practical applications, the FastICA algorithm is adopted in this paper, which adopts a fixed point and Newton iteration; therefore, it has a fast convergence speed.

FastICA assumes that the acquired signal X is preprocessed to obtain a new signal z, where w represents the row vectors in the separation matrix W. The FastICA algorithm maximizes the non-Gaussianity of the vector W^T^X (where T denotes the transpose) by a Newton iteration. From the theory of information theory, we know that negative entropy remains unchanged during invertible linear transformation. In order to obtain a measure for the Gaussian distribution that is zero and always non-negative, negative entropy is the best measure.

**Figure 3:**
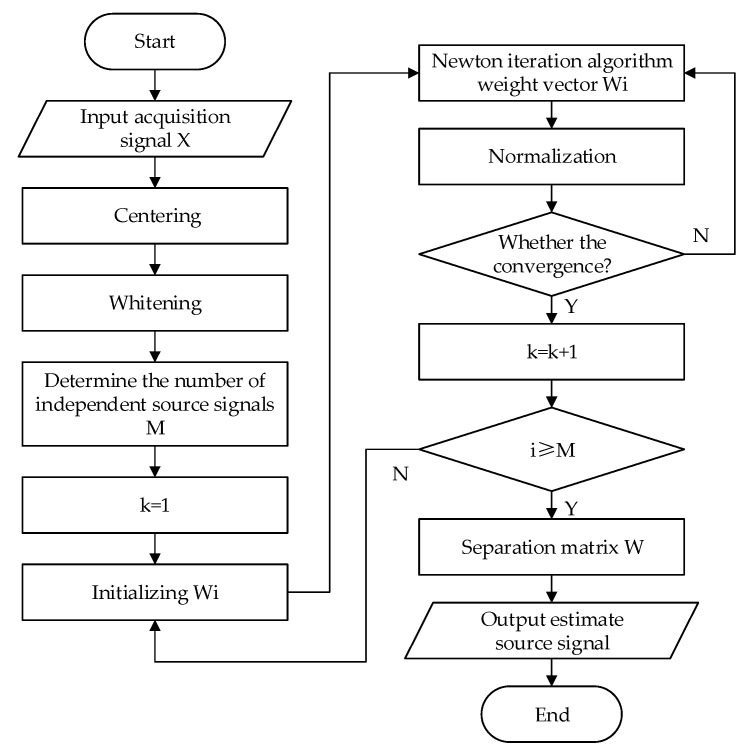
Flowchart of FastICA algorithm.

In general, the observation data are first centralized and whitened to obtain the observation signal with zero mean and unit variance, and then FastICA analysis is performed. The steps of the FastICA algorithm are as follows: (1) Firstly remove the mean of the observed signal X and then whiten it to obtain the variable z. The maximum iteration number N and tolerance error ε are set. (2) Set the initial weight vector w and make the iteration number i = 1. (3) Update W through a Newton iteration and normalize it after each iteration. (4) Suppose i = i + 1, if it does not converge, i.e., i ≤ M and w(i + 1) k − w(i)k > ε, then go back to step 3. The flow chart of the FastICA algorithm is shown in Figure 3.

### 3.3. The Single-Channel MHD Linear Vibration Signal Blind Source Separation Method Based on SSA and FastICA

In real experiments, the output signal of the shaking table is disturbed by a variety of noises when it is received and collected by the MHD linear vibration sensor. Since the noise sources are unknown, in this study, we assume that there are N noise sources, namely X(t) = S(t) + N_i_(t), i = 1, 2, 3, …, n, where, X(t) is the acquisition signal, S(t) is the source signal, and N(t) is the noise signal.

**Figure 4:**
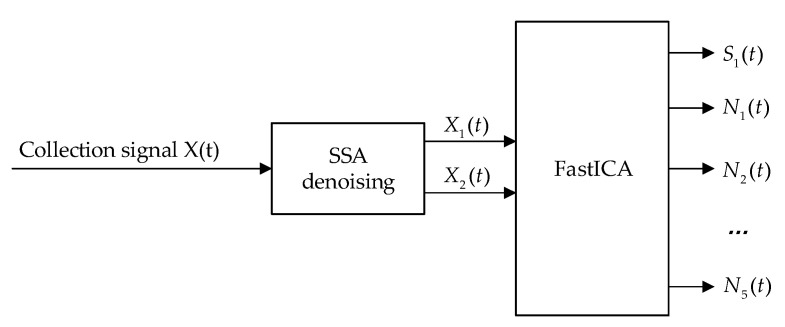
Flow chart of mixed signal separation process based on SSA and FastICA.

The collected linear vibration signal is a mixed signal containing multiple noise signals. The signal-to-noise ratio of such mixed signals is low, and the effect of using ICA directly is not good; thus, the mixed signals need to be denoised first. In this paper, a single-channel line vibration signal separation method based on SSA-FastICA is proposed. Firstly, the output signal of the MHD linear vibration sensor is collected, and the collected signal is a noise-laden mixed signal. Then, the SSA method is preliminarily used to denoise the acquired signal with noise. Finally, FastICA is used to separate the collected signal after denoising, and the unmixed signal was obtained. The separation process of mixed signals based on SSA and FastICA is shown in Figure 4.

The specific implementation steps of single-channel MHD linear vibration signal blind source separation based on SSA-FastICA are as follows:

(1) The MHD linear vibration sensor prototype is placed on the vibration table for vibration detection and its output line vibration signal is collected.

(2) The collected MHD linear vibration signal is processed by SSA, the contribution rate of the eigenvalues of each order component is calculated, and according to the contribution rate of the eigenvalues, the useful signals and noise signals are determined from the components.

(3) The correlation coefficient between each component and the original collected signal is determined, and the threshold of the correlation coefficient is calculated. The relationship between the correlation coefficient and its threshold is used as the criterion to reconstruct the signal components. The reconstructed signal is combined with the original ultrasonic echo signal to form a new multidimensional observation signal, and its dimension is consistent with the estimated number of source signals.

(4) The FastICA algorithm is used to process the newly constructed observation signal to obtain the separated noise signal and useful linear vibration signal.

## 4. Experimental Analysis and Verification

### 4.1. Signal Collected of the MHD Linear Vibration Sensor

**Figure 5:**
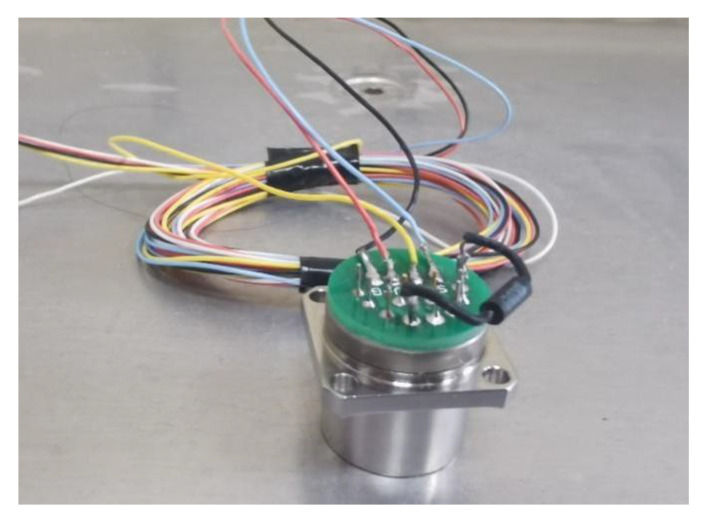
Physical graph of quartz flexible accelerometer.

The MHD linear vibration sensor is a vibration sensor independently developed by the laboratory. In order to measure the accuracy of its actual output linear vibration signal, we chose a quartz flexible accelerometer as the comparison sensor, and its physical diagram is shown in Figure 5. The quartz flexible accelerometer selected in this paper has a high reliability and can be used for dynamic testing. It is a standard vibration sensor with an analog output similar to an MHD line vibration sensor. The parameters of the quartz flexible accelerometer are shown in Table 1.

**Figure 6:**
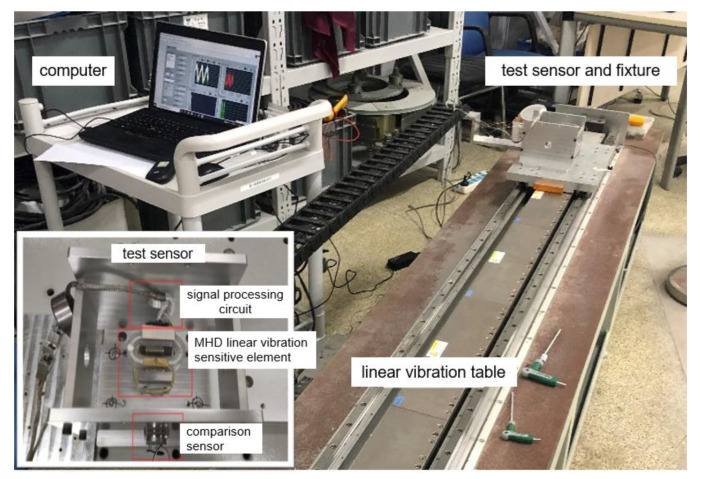
Experimental connection diagram of signal collection of the MHD linear vibration sensor.

The experimental wiring diagram is shown in Figure 6. An MHD linear vibration sensor and quartz flexible accelerometer were placed on the vibration table, and the acquisition card was connected. Finally, the acquired line vibration signal is displayed through the upper computer. Before the experiment, the sensor was fixed with a fixture to prevent position deviation caused by the vibration of the shaking table and affecting the accuracy of the sensor output signal.

**Figure 7:**
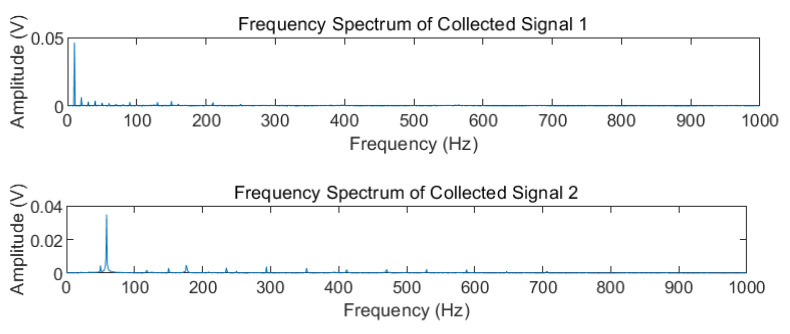

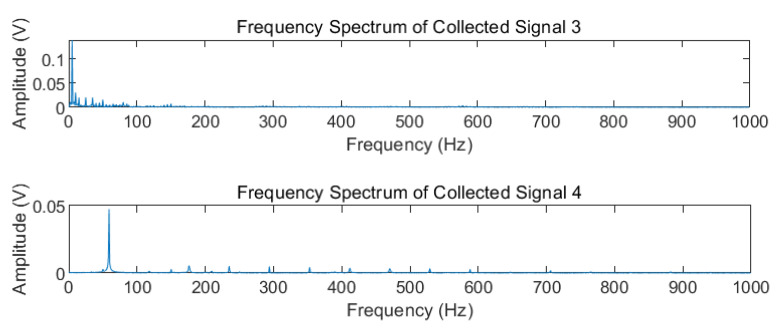
Frequency spectrum of Collected Signal 1–4.

Four groups of experimental data were collected, including 0.6 V, 10 Hz; 0.6 V, 60 Hz; 0.8 V, 5 Hz; and 0.8 V, 60 Hz, denoted as Collected Signal 1, Collected Signal 2, Collected Signal 3, and Collected Signal 4, respectively. The corresponding spectrum analysis of the four groups of collected signals is shown in Figure 7.

### 4.2. The Collected MHD Linear Vibration Signal Is Denoised

For the MHD linear vibration collected signals doped with a variety of noises, their SNR is low and not suitable for direct signal separation. Therefore, the collected linear vibration signals (Collection Signal 1–4) should be preliminarily denoised to obtain a higher signal SNR.

The Collected Signals 1–4 were processed by EEMD, VMD, wavelet threshold denoising, and SSA, respectively, and the useful linear vibration signal components and noise components under the four denoising methods were obtained, respectively.

In order to evaluate the denoising performance of EEMD, VMD, wavelet threshold denoising, and SSA, SNR was selected as the evaluation index of signal denoising. The formula of *SNR* after the preliminary noise reduction in the collected signal is:

(12) $$SNR=10\mathrm{lg}\frac{\sum_{i=1}^{N}s_{i}^{2}\left(t\right)}{\sum_{i=1}^{N}{\left[s_{i}(t)-X_{i}\left(t\right)\right]}^{2}}$$

where *s_i_(t)* represents the source signal, where denoised linear vibration signal is substituted, and *X_i_(t)* indicates the collected signals. When the *SNR* after noise reduction is higher, the separation effect is greater. The *SNR* analysis of these four groups of useful linear vibration signals was carried out, as shown in Table 2.

As seen from the above table, among the collected signals of group 1, 2, 3, and 4, the signal after SSA processing has a higher SNR than the signal after EEMD, VMD, and wavelet threshold denoising processing, and the SNR of the signal after SSA processing is 1.6 to 32 times that of the other three kinds. Therefore, it can be seen that SSA technology is a better choice for pre-denoising the collected signals when comparing the SNR improvement of the four groups of collected signals after processing.

### 4.3. Collection Signal Noise Reduction and Re-Separation

For mixed signals with a low SNR, after SSA is first used to reduce noise and improve the SNR, FastICA can be directly used to separate the collected signals with high SNR, and independent source signals, i.e., useful linear vibration signals, can be obtained. Blind signal separation was performed in four groups of collected signals to obtain six separated signals: separated signal 1, separated signal 2, separated signal 3, separated signal 4, separated signal 5, and separated signal 6. The similarity coefficient between each group of separated signals and the corresponding collected signals is shown in Table 3.

**Figure 8:**
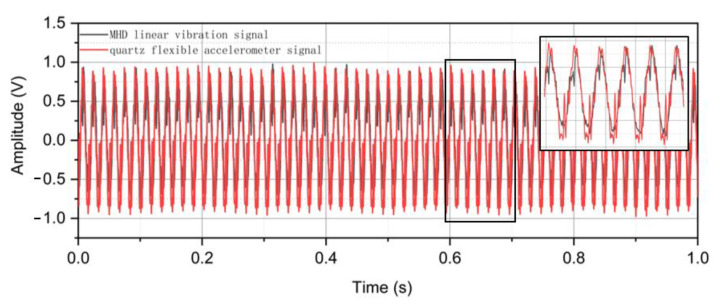
Comparison of output signals of quartz flexible accelerometer and linear vibration signals of MHD after SSA-FastICA processing.

The four groups of signals with the highest similarity coefficients among the decomposed signals are found, as shown in Table 4. These four groups of signals are the line vibration signals obtained by processing the four groups of collected signals by the SSA-FastICA algorithm. It is observed that the output signals of the quartz flexible accelerometer are essentially the same as the decomposed linear vibration signals waveform, as shown in Figure 8. In contrast, in the quartz flexible acceleration sensor and our experimental test sensor, the output voltage signals amplitudes are different. Therefore, it is more appropriate to measure the output signal of the MHD linear vibration sensor and quartz flexible accelerometer by signal similarity. The similarity of the output signals is more than 98%. It can be seen that the single-channel linear vibration signal blind source separation method based on SSA-FastICA can effectively extract standard linear vibration signals. In order to test the repeatability of the sensor, multiple sets of data of the MHD linear vibration sensors at different times on different dates are collected. The formula for the standard error (SE) [31] is shown below:

(13) $$SE=\sqrt{\frac{1}{n-1}\sum_{i=1}^{n}(X_{i}-\bar{x}{)}^{2}}$$

(14) $$\bar{x}=\frac{1}{n}\sum_{i=1}^{n}X_{i}$$

where *X_i_* represents the collected signal segment and

$$\bar{x}$$

represents the average of all signal segments.

**Figure 9:**
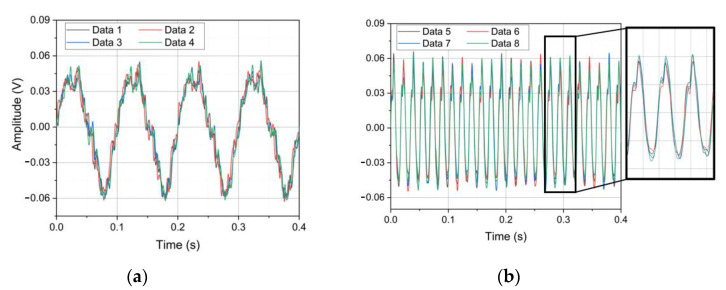
(**a**) Random four signal segments at 10 Hz; (**b**) Random four signal segments at 60 Hz.

The SE of the data segments of 10 Hz and 60 Hz is calculated, respectively. Eight data segments of 10 Hz and 60 Hz are randomly selected, as shown in Figure 9. The signal average SE of the quartz flexible accelerometer is 0.02348 mV at 10 Hz and 0.56456 mV at 60 Hz. The signal average SE of the MHD linear vibration sensor is 0.00620 mV at 10 Hz and 0.03684 mV at 60 Hz. After SSA-FastICA algorithm processing, the average SE of signals of the MHD linear vibration sensor is 0.00420 mV at 10 Hz and 0.00392 mV at 60 Hz. Therefore, it can be considered that the MHD linear vibration sensor has good repeatability.

## 5. Conclusions

Based on the theoretical basis of MHD, this paper designs and prepares a prototype of a runway-type fluid ring type linear vibration sensor, and samples and extracts the output linear vibration velocity signal. Since the acquired signals belong to mixed signals containing multiple complex noises, a single-channel linear vibration signal separation method combining SSA and FastICA is proposed through the study of blind signal extraction techniques, and the effectiveness of the method is verified by experimental results. The following conclusions are obtained:

1. The self-made MHD linear vibration sensor possesses the advantages of no mechanical wear between internal firmware and no additional power supply. However, its limitation is that it is not suitable for constant speed measurement.
2. For mixed signals containing multiple narrowband noise signals, there is a large difference between the signals directly separated by the ICA algorithm and the source signal. For the collection signal with more noise, noise reduction is needed to improve the SNR.
3. Compared with the processing methods of EEMD, VMD, and wavelet threshold noise reduction, the SSA algorithm can effectively suppress the narrowband periodic interference and white noise in the mixed signal, improve the SNR of the acquired signal, and effectively retain the useful information of the signal while reducing noise.
4. The proposed SSA-FastICA algorithm can effectively solve the problem of blind source separation and the extraction of the single-channel MHD linear vibration signals to minimize the dynamic measurement error. Thus, it shows that the SSA-FastICA method can be effective in single-channel MHD linear vibration signal separation and processing.

## Figures and Tables

**Table 1:** Parameter list of quartz flexible accelerometer.

Parameters	Parameters Setting
0 g 4-h short-term stability	≤20 μg
1 g 4-h short-term stability	≤20 ppg
Partial value synthetic repeatability	≤40 μg
Scale factor synthesis repeatability	≤50 ppg
Comprehensive repeatability of nonlinear coefficient	≤±20 μg/g^2^
Partial temperature coefficient	≤±30 μg/°C
Scale factor temperature coefficient	≤50 ppg/°C
Noise	≤4 mV

**Table 2:** Table of signals SNR comparison after EEMD, VMD, wavelet threshold denoising, and SSA processing.

Denoising Algorithm	Collected Signal 1	Collected Signal 2	Collected Signal 3	Collected Signal 4
EEMD	10.7121	0.7196	4.0292	0.8049
VMD	34.9907	10.1769	17.5607	11.3256
Wavelet Threshold Denoising	33.1645	11.3181	32.2446	11.1439
SSA	58.6428	23.1137	52.4412	21.5621

**Table 3:** Similarity factor between the separated signals and the collection signals 1–4.

Serial Number	Collected Signal 1	Collected Signal 2	Collected Signal 3	Collected Signal 4
Separated Signal 1	2.61%	13.67%	1.68%	9.02%
Separated Signal 2	98.45%	98.82%	2.59%	99.26%
Separated Signal 3	3.20%	4.60%	16.31%	4.53%
Separated Signal 4	11.98%	1.54%	−6.35%	0.88%
Separated Signal 5	11.86%	−3.50%	6.97%	3.89%
Separated Signal 6	1.54%	2.20%	98.15%	−4.93%

**Table 4:** Similarity factor (max) between the separated signals and the Collected Signals 1–4.

	Collected Signal 1	Collected Signal 2	Collected Signal 3	Collected Signal 4
Similarity Factor (max)	98.45%	98.82%	98.15%	99.26%
